# A High-Throughput Cidality Screen for Mycobacterium Tuberculosis

**DOI:** 10.1371/journal.pone.0117577

**Published:** 2015-02-18

**Authors:** Parvinder Kaur, Anirban Ghosh, Ramya Vadageri Krishnamurthy, Deepa Gagwani Bhattacharjee, Vijayashree Achar, Santanu Datta, Shridhar Narayanan, Anand Anbarasu, Sudha Ramaiah

**Affiliations:** 1 AstraZeneca India Private Limited, Bangalore, Karnataka, India; 2 Department of Microbial and Molecular Systems, KU Leuven, Kasteelpark, Arenberg 20, 3001 Leuven, Belgium; 3 Gangagen Biotech Private Limited, Bangalore, Karnataka, India; 4 Cellworks India, Bangalore, Karnataka, India; 5 Medical & Biological Computing Laboratory, School of Biosciences & Technology, VIT University, Vellore, Tamil Nadu, India; University of Padova, Medical School, ITALY

## Abstract

Exposure to *Mycobacterium tuberculosis* (Mtb) aerosols is a major threat to tuberculosis (TB) researchers, even in bio-safety level-3 (BSL-3) facilities. Automation and high-throughput screens (HTS) in BSL3 facilities are essential for minimizing manual aerosol-generating interventions and facilitating TB research. In the present study, we report the development and validation of a high-throughput, 24-well ‘spot-assay’ for selecting bactericidal compounds against Mtb. The bactericidal screen concept was first validated in the fast-growing surrogate *Mycobacterium smegmatis* (Msm) and subsequently confirmed in Mtb using the following reference anti-tubercular drugs: rifampicin, isoniazid, ofloxacin and ethambutol (RIOE, acting on different targets). The potential use of the spot-assay to select bactericidal compounds from a large library was confirmed by screening on Mtb, with parallel plating by the conventional gold standard method (correlation, r^2^ = 0.808). An automated spot-assay further enabled an MBC90 determination on resistant and sensitive Mtb clinical isolates. The implementation of the spot-assay in kinetic screens to enumerate residual Mtb after either genetic silencing (anti-sense RNA, AS-RNA) or chemical inhibition corroborated its ability to detect cidality. This relatively simple, economical and quantitative HTS considerably minimized the bio-hazard risk and enabled the selection of novel vulnerable Mtb targets and mycobactericidal compounds. Thus, spot-assays have great potential to impact the TB drug discovery process.

## Introduction

Tuberculosis continues to pose a major threat to mankind at a global level [1]. Existing anti-TB drugs have almost become obsolete because of poor compliance and the emergence of multiple drug resistance (MDR), extensive drug resistance (XDR) and total drug resistance (TDR) [1]. Furthermore, the discovery of novel anti-TB agents has been hampered by the inherently slow growing (doubling in 16–24 hrs.) and highly infectious nature of *Mtb*. The aerosol transmissibility of the infection [2,3] confines TB research to highly specialized bio-containment BSL3 facilities [4]. It remains a challenge to speed up the drug discovery process to identify new bactericidal and sterilizing compounds and provide a definitive cure for TB patients.

Whole-cell screening (WCS) assays are fueling the early discovery of new lead compounds in various disciplines from infections to cancer [5]. There are many examples of chemical leads emerging purely from WCS strategies and compounds that have progressed to the clinic (e.g., TMC 207 or Bedaquiline, an anti-TB compound) [6]. Early discovery programs are increasingly adopting WCS strategies because of the demonstrated speed, feasibility of high-throughput screening (HTS), miniaturization [7] and enhanced sensitivity of reporter gene signal amplification assays in recombinant strains either in Mtb or fast-growing surrogate mycobacteria [8,9,10]. Although these microbes grow faster, the differences in susceptibility, drug penetration and target differences in surrogates [10] may lead to higher attrition rates. The same is true for faster readouts [11].

Generally, most available HTS methods select only static hits in minimum inhibitory concentration (MIC)-based assays, but these tests are based on only Mtb growth inhibition. Among the available screening systems, BACTEC-TB 460 and MGIT-TB 960 are quantitative but not capable of high-throughput analysis, and they require expensive specialized instruments and media. The high-throughput screening assays, *viz*. the Microplate Alamar Blue Assay (MABA) and the REsazurin-based Microplate Assay (REMA), are high throughput, but these assays merely provide MIC information and do not predict the cidal capacity of the test compounds. In addition, there are reporter-based assays, such as luciferase (*lux*), or green fluorescent proteins (GFP), that require modifications for each strain [12]. Therefore, it is important to develop methods that can detect the anti-TB cidality potential or minimum bactericidal concentration (MBC) of inhibitors directly on Mtb during early screening. Bactericidal screening would have greater physiological relevance because ‘cidality’ is the real indicator of bacterial death.

Detecting the bactericidal activity of compounds requires the plating and enumeration of survivors (bacterial colonies) [13] after determining the MIC [14]. However, only a few selected compounds can be tested for MBC studies because of several challenges. It is technically very laborious and low throughput to plate out various dilutions of drug-treated samples and then identify the minimal concentration that kills the bacteria (MBC ≥2 log_10_ reduction in cfu). It takes a minimum of 3 weeks to grow readable colonies of Mtb. Therefore, new approaches for selecting bactericidal anti-TB compounds and essential targets are urgently required to accelerate the drug discovery cascade rather than adding cidality through retrospective chemical modifications [15]. The objective of this study was to validate and apply a BSL-3-compliant, high-throughput spot-assay to enumerate Mtb survivors following microbicide treatment using either chemicals (compounds) or genetic manipulation (e.g., by AS-RNA, one of the novel approaches to select bactericidal targets [16,17]).

Hence, we hypothesized and validated a bio-safety-compliant, high-throughput ‘spot-assay’ for Mtb cfu enumeration. Applying the spot-assay to kinetic screens in the BSL3 facility extended its utility for selecting tuberculocidal compounds and cidal targets in a bio-safe and efficient manner.

## Materials and Methods

Understanding the complexity of HTS logistics, especially in a BSL3 facility, is the key to screening programs. We incorporated several automations, e.g., assay plate preparation (Biomek-Fx, Beckman Coulter Biomek FX Workstation), ready-to-use Mtb culture stock preparation (frozen, enumerated), culture dispensation (Multidrop-Combo), a results readout (Spectramax^PLUS384^), agar dispensation (Automed media dispenser), MBC culture spotting on 24-well agar plates (12 channel/multichannel Pipetman and Biomek-2000 system housed in a customized bio-safety cabinet) and data upload (Hbase, which is sophisticated software for data analysis).

### Bacterial strains, media, chemicals and reagents

Mtb H37Rv ATCC27294 wild type (WT); sensitive (ATCC28218, ATCC35811, Erdman, Beijing, Harlingen, CDC1551, HN878, SA161, SA310, TN14149, SJ396, DKU76, 97A, 211 and 220) and single drug-resistant (SDR) clinical strains of Mtb (ATCC35820, ATCC35822, S6570, I2253 and O12119) and Msm ATCC607 were obtained from the AstraZeneca India (AZI) strain collection. All chemicals and reference drugs, namely, rifampicin, isoniazid, ofloxacin, ethambutol, tetracycline and streptomycin, were procured from Sigma Chemical Co. Drug solutions were prepared in dimethyl sulfoxide (DMSO), Milli-Q water or as otherwise specified. The bacterial culture media, which were Middlebrook-7H9 broth (Cat#271310) for determining the minimum inhibitory concentration (MIC) and 7H10 agar (Cat#254521) for determining the minimum bactericidal concentration (MBC), were procured form Becton Dickinson-Difco. The plastic ware used was as follows: 384-well assay plates for the MIC studies (Greiner Inc., Cat# 781186) and Petri plates and multi-24-well plates (Costar#3524) for the MBC studies.

### A schematic of the initial standardization and validation

**Fig 1:**
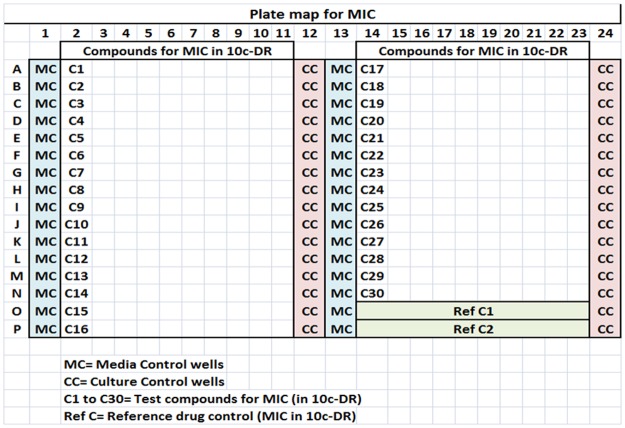
A plate map for the MIC in a 384-well plate. A total of 30 test compounds/plate (2 compounds/row) could be tested as a 10-concentration dose response (10c-DR as A2–11 to P2–11 and A14–23 to N14–23). **Quality controls (QC)**: Columns 1 and 13 = media controls (MC), columns 12 and 24 = culture controls (CC). The 31^st^ and 32^nd^ rows = reference drug controls (e.g., isoniazid in rows O&P14 to O&P23) and rows MN14 to MN23 were used for additional reference drug QC, if required. Culture addition was performed with the Multidrop-Combi liquid handling system, and incubation was performed at 37°C for 3–4 days (Msm) and 3–4 weeks (Mtb). Post-incubation, the MIC data were recorded using a spectrophotometer (Spectramax^384Plus^) at O.D._600nm_.

The spot-assay began with a 384-well culture plate (MIC/assay plate) as the first step. The MIC was setup per the CLSI [14] guidelines. In brief, for MIC testing, the compounds or the reference drugs Rifampicin, Isoniazid, Ofloxacin and Ethambutol (RIOE) were dispensed as serial 2-fold dilutions from the highest to the lowest concentrations (column 2 through column 11 and column 14 through column 23, Fig. 1) and used to determine the 10-concentration dose response (10c-DR); maximum start concentrations of RIOE of 128, 16, 4 and 8μg/ml, respectively, were tested for Msm. The culture was added at a final start inoculum of approximately 3–7x10^5^ cfu/ml, and after incubation of the plates for 3 days at 37°C, the growth of the colonies was monitored at O.D._600nm_ using a spectrophotometer (Spectramax^PLUS384^), and the data were recorded. The lowest drug concentration that inhibited ≥80% growth was considered the MIC. Following the completion of an MIC assay, the cultures were aspirated and spotted on to 24-well agar plates to enumerate the cfus.

**Fig 2:**
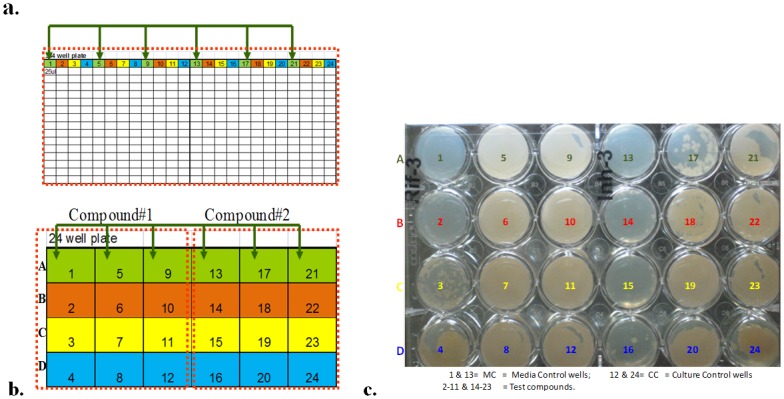
The schematics of spotting from an MIC plate to a 24-well plate. **a**. Culture aspiration from a MIC plate using filter-tips fixed at alternate positions of a 12-channel Pipetman. **b**. **Twenty-four-well plate**: Row-wise dispensing into a 24-well plate. Two compounds/row, with a 10c-DR each: e.g., Row A: Compound#1 = 2–11 wells and Compound#2 = 14–23 wells (1 & 13 = media control, 12 & 24 = culture control). One plate of 24 wells = data for 2 compounds (10c-DR) for 1 row of MIC plate: The left half of the 24-well plate contains the 1^st^ compound (#2 to 11), and the right half contains the 2^nd^ compound (#14 to 23). **c. A picture of a spot-assay in a 24-well plate**: colony morphology and countable colonies (e.g., rifampicin on the left half and isoniazid on the right half of the plate).

Multi-well culture spotting from the MIC plate wells to the 24-well agar plates was performed with a multichannel Pipetman (12-channel, Fig. 1, 2) and later using a Biomek-2000, a robotic liquid handling system. After spotting the culture, 24-well agar plates were incubated at 37°C in a CO_2_ incubator for 3–4 days for Msm and for 3–4 weeks in the case of Mtb.

1. The minimum culturable volume for spotting on 24-well agar beds

An initial optimization was performed with fast-growing surrogate Msm ATCC607. A detailed study was performed with a log-phase culture of Msm by spotting 100μl of culture (100μlX1 plate, 50μlX2, 25μlX4, 12.5μlX8, 6.25μlX16, 3.125μlX32 and 1.56μlX64 plates) directly or with doubly diluted cultures on 24-well plates and on Petri plates, in parallel, to enumerate the dispensed cfu/ml. The total plated volume was kept constant at 100μl (e.g. 1.56μl plated on 64 plates as 1.56μlX64 = 100μl, another 100μl as 3.12μlX32 = 100μl, and so on until 100μlX1) for precision, uniformity and comparison across cfus; however, the volumes were divided into different numbers of plates.

2. Drug carry-over assay

i) Bioassay

Five drugs, namely, rifampicin (R), streptomycin (S), ofloxacin (O), tetracycline (T) and isoniazid (I), were absorbed into 24-well agar plates for 4 hrs. at 37°C at the maximum tested concentrations (RIOST tested at 64, 8, 8, 2 and 16μg/ml concentrations) along with the quality control (QC). Drug-impregnated agar blocks were punched from these plates and tested by diffusion assays on a lawn culture of Msm. The zone of inhibition was recorded after incubation.

ii) Cfu enumeration

Isoniazid, a bactericidal drug, was used to test this concept because it is a mycobacteria-specific inhibitor. Hence, this drug was used as a QC drug to ensure that no non-mycobacterial/ rapid-growing bacterial contamination occurred during the assay. The Msm culture was exposed to concentrations in a range of 16–0.03μg/ml. Twenty-five microliter aliquots from the control and isoniazid-containing wells were transferred immediately after drug exposure and plated, in parallel, on 24-well and Petri plates. Following the incubation of these agar plates, the cfus were enumerated, and the data were plotted and compared to evaluate drug carry-over, if there was any.

3. Spot-assay in *M. smegmatis* using the reference drugs Rifampicin, Isoniazid, Ofloxacin and Ethambutol (RIOE)

The MIC was the starting point for the spot-assay, and it was setup according to the CLSI guidelines [14] for 384-well plates, as described above. Spot-assay method validation was performed by using reference drugs (RIOE) on the fast-growing surrogate mycobacteria- Msm ATCC607 to obtain faster results and to incorporate rapid modifications. The spot-assay for MBC was performed by accurately dispensing 25-μl culture volumes onto 24-well agar plates in parallel with the conventional Petri plates (85-mm diameter) for comparison (Fig. 2a, b). The entire set of 15–20 plates was swirled together instead of laboriously culture-streaking them onto individual plates. The plates were incubated at 37°C for 3 days, and the cfus were enumerated. Three independent experiments (inter-experiment variation) with triplicate sets (intra-experiment variation) for each experiment were compared by a parallel conventional plating method. The MBC was the concentration at which the bacterial cfu was reduced by ≥2 log_10_ from the starting inoculum (~3–7X10^5^ cfu/ml). The automation was built at every step to increase the throughput and robustness.

4. Spot-assay of *M. tuberculosis* using the reference drugs RIOE

The methods, protocols and test conditions validated in Msm were applied to Mtb for the final proof of concept and implementation. The MIC of Mtb H37Rv ATCC27294 was determined by using reference drugs (RIOE at 2, 2, 8 and 32μg/ml respectively). The spot-assay was performed on 24-well plates in triplicate to build consistency. The plates were incubated at 37°C for 3–4 weeks, and the cfu data were recorded (Fig. 2b).

5. Spot-assay automation in Mtb (Biomek-2000)

The primary objective of this screen was to develop a cidality screen for testing sensitive and MDR strains of Mtb under BSL3 containment. Once the whole process layout had been established, automation was incorporated at the most crucial culture dispensing stage. Because the Biomek arm moves horizontally, the culture-dispensing schematic was changed from vertical (**row-wise** i.e. from row A of a 384-well plate to row A of a 24-well plate, and so on) to horizontal (**column-wise** i.e. from column 1 of a 384-well plate to column 1 of a 24-well plate, and so on). The Biomek was housed in a customized bio-safety cabinet to contain the aerosols generated during the culture transfer procedures.

6. Screening a compound library on Mtb

A set of 250 compounds from a single-point inhibition WCS was taken up for MIC confirmation in a 10c-DR (test compounds from 60μM to 0.1μM and the reference control drug isoniazid from 16–0.031μM) and subsequently for MBC by spot-assay using a Biomek-2000 for culture transfers. No manual culture spreading was required, which considerably reduced the time and the generation of aerosols in the bio-safety cabinet. A set of 20–25 plates was swirled, instead of streaking; the plates were incubated at 37°C for 3–4 weeks under appropriate bio-safety conditions. A schematic card was prepared for rapid data assessment. The data were recorded once growth/colonies appeared on the culture control wells.

7. MBC_90_ studies in *M. tuberculosis*

Following the Biomek automation in the BSL3 setup with the WT strain for Mtb, we applied the method to perform MBC_90_ studies. The MIC against *M. tuberculosis* clinical isolates (both drug-susceptible and single-drug-resistant strains) was determined using the same protocol as described above. The effects of a select set of 4 compounds from an advanced project were tested on a large panel of Mtb strains (n = 20), including sensitive, SDR, laboratory and clinical isolates, using spot-assays under BSL3 containment.

8. Applying the spot-assay to kinetic screens in Mtb

The final objective of the high-throughput spot-assay was its application to kinetic screens for selecting cidal targets as follows: 1) first, we measured the **killing kinetics** of rifampicin at 0, 3, 7 and 14-day intervals, and the results of the spot-assay were compared *vs*. the conventional method reported earlier [18] to check the feasibility; 2) finally, the spot-assay was applied to the **survival kinetics** of Mtb AS-RNA gene silencing to differentiate cidal *vs*. static targets. To achieve this result, the AS-RNA construct of Mtb polyphosphate kinase (*ppk*) from our previous study was used [16,17]. A concept validation of the spot-assay for the AS-RNA survival kinetics in Mtb was performed using ppk-AS in triplicate. The outcome was compared against the conventional data that was reported earlier [17]. The cfu data were plotted in GraphPad Prism ver.4.0 to analyze the kinetic graphs. To strengthen the utility of spot-assays for kinetic screens, we selected 2 more targets, namely, phosphopantetheine adenylyl transferase (*coaD)* and uridylate kinase (*pyrH)*. The *coaD* and *pyrH* genes of Mtb were freshly cloned, and the survival kinetics of both AS cultures were measured in triplicate by using spot-assays at days 0, 3, 10, 17, 28 and 35. At the end of an incubation period of 3–4 weeks at 37°C, bacterial survivors were enumerated for cfu and the data were plotted *vs*. the conventional plating data for comparison.

## Results

1. The minimum culturable volume for spotting on 24-well agar beds

**Fig 3:**
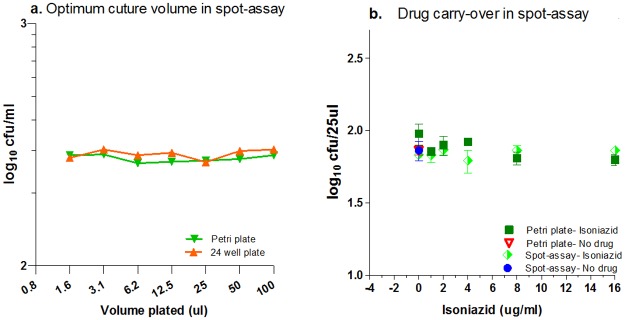
Optimisation of assay conditions. Initial assay optimization was done in Msm: **a. Minimum culturable volume in Msm**: Msm culture was dispensed in 1.56-μl aliquots (1.56μlX64) multiple times, up to 50μl twice (50μlX2), on 24-well agar beds and conventional plates. The sum of the cfu obtained for each volume was plotted. The parallel lines of the cfu data plotted against the plating volumes confirmed that there was no variation in the net bacterial counts obtained from plating different volumes. Hence, the 25-μl volume was selected. **b. Drug carry-over assay in Msm**: Untreated and isoniazid-treated samples yielded a similar number of cfus. Isoniazid exposure yielded similar bacterial counts from both the conventional and 24-well spot-assay over a range of concentrations up to 16 μg/ml.

A good concordance of the cfu data was observed, whether the total 100μl volume was plated on one plate, or divided on multiple plates. The total number of colonies obtained from 100μl of plated volume or, e.g., the sum of colonies from 1.56μl plated in 64 wells (or 3.12μlX32 = 100μl and so on), was similar, suggesting that any volume within this range could be optimal for spotting (Fig. 3a). Parallel cfu data obtained from conventional Petri plates corroborated the results of the 24-well spot-assay (Fig. 3a). Hence, a convenient volume of 25μl was selected for spotting onto 24-well agar plates for all studies.

2. Drug carry-over

The MICs of the reference drugs RIOE on Msm ATCC607 strain were 16, 2, 0.125 and 1μg/ml respectively. The next step of the validation was to rule out any drug carry-over to 24-well agar beds. i) Bioassay: No zone of inhibition was observed on Msm lawns in Petri plates with any of the five drugs (RIOST), even when tested at the highest concentrations (64, 8, 8, 2 and 16μg/ml). ii) cfu enumeration: No differences in the cfu outcomes from isoniazid-exposed and ‘no-drug’ culture control samples or those plated on either 24-well beds or conventional Petri plates were observed (Fig. 3b). Hence, no drug carryover was observed in the 25-μl aliquots that were transferred to set up the spot-assay.

3. Spot-MBC in Msm and Mtb using the reference drugs RIOE

**Fig 4:**
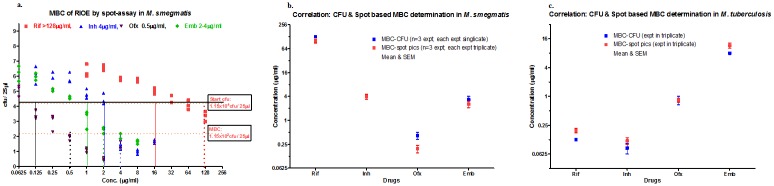
Spot-assay correlation in Msm. To check suitability of the assay. **a**. **MBC correlation** showing inter-assay variability (n = 3) in the cfu-based assay in a 25-μl volume in Msm using RIOE. **b**. **Correlation of spot *vs*. cfu in Msm** for RIOE. MBC values were deduced from this finding (**a**.) and were compared with the data obtained by the conventional method. The spot-assay MBC of four RIOE reference drugs on Msm was compared with the standard cfu-based plating assay. Cfu-based MBC: Minimum concentration that yielded a ≥2 log_10_ reduction from the starting cfu. Spot-based MBC: Minimum concentration that resulted in no growth (NG) or countable colonies (upto 30 colonies). The data for all four drugs correlated well for both methods (Fig. 5b). **c**. **Correlation of spot *vs*. cfu of Mtb** for RIOE. This method was subsequently replicated in Mtb for all four RIOE reference drugs.

The cfu results from three independent experiments in the Msm spot-assay, each of which was performed in triplicate, were plotted, and the MBC (≥2log_10_ cfu reduction) was extrapolated from the starting cfu as shown in Fig. 4a. All four reference drugs exhibited a good 10c-DR (Fig. 4a). The method also confirmed the MIC, as indicated by the vertical solid lines (Oflx 0.125μg/ml, Emb 1μg/ml, Inh 2μg/ml and Rif 16μg/ml) in Fig. 4a. The MBC was taken as a ≥2log_10_ cfu reduction from the starting cfu and is represented by dotted vertical lines. The comparative MBC of RIOE against Msm was Oflx (0.5–1μg/ml), Emb (2–4μg/ml), Inh (4–8μg/ml) and Rif (≥128μg/ml). The starting cfu was 1.5X10^4^/25μl (4.6X10^5^/ml), and the MBC, shown as a ≥2log_10_ reduction in 1.15X10^2^cfu/25μl (3.45X10^3^cfu/ml), is indicated by a horizontal dotted line. The MBC data obtained from the spot-assay correlated well with the conventional plating data for all four drugs (RIOE, Fig. 4b). The spot-assay-based data on all four drugs were consistent, as supported by the minimal variation in three independent experiments. The data exhibited a good correlation with the standard cfu-based MBC method (Fig. 4b). The initial validation method for Msm, with consistent inter- and intra-assay robustness, suggested high confidence in the spot-assay. Hence, the spot-assay validated for Msm was implemented for Mtb. The spot-assay could be successfully replicated in Mtb (a single experiment, in triplicate) with all 4 RIOE drugs and data plotted (mean and SEM) (Fig. 4c).

After manually completing the validation, an automation setup was established in the BSL3 facility. A Biomek-2000 liquid handling system was calibrated and housed in a customized bio-safety cabinet in the BSL3 facility to conduct experiments on the Mtb pathogen. This system enabled the handling of multiple Mtb strains (sensitive/drug-resistant Mtb) for testing large-compound libraries in the BSL-3 setting.

4. A spot-assay for screening the compound library in Mtb

**Fig 5:**
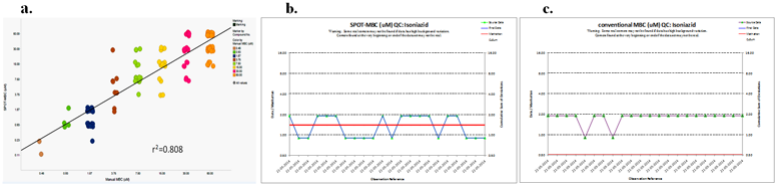
Spot-assay correlation in Mtb and the Manhattan analysis. The spot-assay was replicated in Mtb for RIOE. **a. An MBC correlation between the spot-assay and the conventional method**: A strong positive correlation (r^2^ = 0.808). **b & c: The Manhattan analysis** of isoniazid-treated reference controls exhibited variation within the 2-fold in the spot-assay (**b**) and conventional MBC methods (**c**).

The high-throughput spot-assay was used to determine the MBC of a set of 250 compounds in parallel with the standard plating method in the BSL3 facility. As a result, 179/250 (71%) compounds exhibited MBC according to the gold-standard cfu plating method, and 189/250 (75%) exhibited MBC according to the spot-assay. There was consistency in the MBC detected in the two methods because 173 (91.5%) were MBC-positive according to both methods. The MBC response was distributed across the entire 10c-DR range (0.1μM to 60μM); however, most compounds (~80%) were distributed between 7.5μM and 60μM. An analysis using the Spotfire software revealed an excellent positive correlation (r^2^ = 0.808) between the spot and manual MBC (Fig. 5a). Among the MBC-positive compounds, 99.2% showed good consistency, with variations limited to within 4-fold (only 2 compounds exhibited an 8-fold variation).

A critical evaluation of isoniazid controls from each assay plate was performed using Manhattan analysis to strengthen the validation. The results suggested highly consistent and reproducible MBC values (within 2-fold variations) for isoniazid across all of the plates that were screened (Fig. 5b,c). The QC data of the isoniazid drug confirmed the robustness of the spot MBC assay.

5. The application of the spot-assay to MBC_90_ studies

The adoption of automation in the BSL3 facility enabled the opportunity to screen even a larger panel of drug-sensitive and drug-resistant Mtb clinical isolates in a high-throughput manner. The liquid handling system enhanced the speed, precision and bio-safe handling of drug-resistant pathogens. A set of 4 selected bactericidal compounds were tested against 20 Mtb strains and simultaneously enabled a rapid and precise determination of the MBC_90_ against multiple strains (Table 1).

**Table 1:** MBC_90_ studies: MBC_90_ testing on *M. tuberculosis* (sensitive and drug-resistant isolates) is possible.

MBC_90_ studies for project compounds (MBC μg/ml)						
S.No.	Mtb strains	Cmpd.#1	Cmpd.#2	Cmpd.#3	Cmpd.#4	INH
1	Mtb-Rv	2	2	2	2	1μg/ml
2	Mtb-JCT	2	4	16	32	
3	Mtb-ERD	4	2	16	8	
4	Mtb-BEIJI	8	16	32	32	
5	Mtb-HARL	8	4	64	64	
6	Mtb-DKU-76	2	2	4	4	
7	Mtb-DKU-97A	0.5	0.3	0.5	16	
8	Mtb-DKU-211	8	8	16	16	
9	Mtb-DKU-220	1	1	2	2	
10	Mtb-35811	32	16	8	16	
11	Mtb-TN14149	4	4	8	16	
12	Mtb-HN878	4	8	16	16	
13	Mtb-SA161	4	4	4	16	
14	Mtb-SA310	32	16	4	32	
15	Mtb-SJ396	8	8	16	4	
16	Mtb-35820	4	16	16	32	
17	Mtb-S6570	8	8	4	8	
18	Mtb-I35822	8	8	32	16	
19	Mtb-I2253	1	2	8	4	
20	Mtb-O12119	4	4	4	8	
**Data**	**MBC_90_ value**	**8**μ**g/ml**	**16**μ**g/ml**	**32**μ**g/ml**	**32**μ**g/ml**	**1**μ**g/ml**

The spot-assay enabled the reporting of MBC_90_ data in a high-throughput manner for the first time in the literature. The validation of various steps in the method and of the necessary instrumentation led to the development of an efficient, ergonomic and economical (EEE) MBC screening tool that was compliant with bio-safety (BSL3 level) containment constraints and can lead to the faster identification of bactericidal compounds for the slow-growing pathogen Mtb, right from its earliest discovery.

6. Applying the spot-assay to kinetic screens

**Fig 6:**
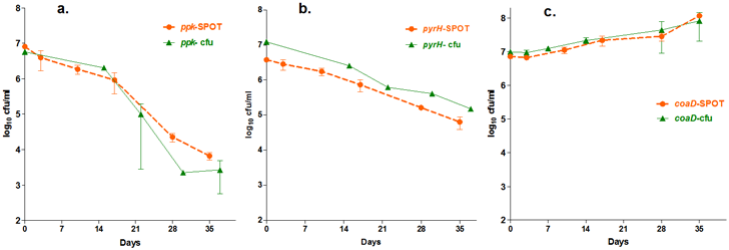
Kinetic screen-spot vs. conventional assay. The survival kinetics of all three Mtb-AS target genes (*ppk*, *pyrH* and *coaD*) demonstrated quantitative cidality in real time on different days. All findings are reproducible and exhibit comparable overlapping graphs (two-tailed P values <0.001 for all three AS data), with a strong positive correlation (ppk r^2^ = 0.693, *pyrH* r^2^ = 0.920 and *coaD* r^2^ = 0.856).

The killing kinetics of rifampicin by spot-assay showed agreement with the conventional assay data that was reported earlier (data not shown) [18]. This milestone encouraged the further application of the spot-assay toward our final goal of performing survival kinetic screens for Mtb by using **AS-RNA** as a tool for target validation. The survival kinetics data obtained by spot-assay for Mtb ppk-AS demonstrated overlapping graph trends *vs*. the conventional assay and showed a >2log_10_ reduction in the cfu on day 35. Similar confirmatory kinetics results were observed for *coaD* and *pyrH* when subsequently tested. The spot-assay was able to confirm the data for both cidal (*ppk* and *pyrH*) and static (*coaD*) targets in the kinetics screen. Based on these data, the target cidality could be rated as *ppk>pyrH*, and the *coaD* target was confirmed as static by both methods. These target essentiality data matched those of previous reports [19]. This concordance of spot-assay results with the gold-standard plating method confirmed the strength of the assay. The spot-assay readout of AS cultures (*ppk*, *coaD* and *pyrH*) exhibited robust overlapping survival kinetic profiles *vs*. the conventional plating method (Fig. 6) across time intervals [16,17], with a strong positive correlation (two-tailed P values, <0.001 for all three AS data sets; and Pearson’s correlation for *ppk* r^2^ = 0.693, *pyrH* r^2^ = 0.920 and *coaD* r^2^ = 0.856). The results confirmed the applicability of the spot-assay and its precision in complex kinetic screens, whether they involved genetically manipulated cultures or were perturbed by compound inhibition in a high-throughput manner.

## Discussion

*Mycobacterium tuberculosis* is an extremely successful pathogen that has developed the ability to hide, undergo dormancy, and reactivate in the host. The successful eradication of TB requires cidal therapeutics to eliminate the various physiological forms of the bacteria hiding in privileged niches. Additionally, the highly infectious and slow-growing nature of Mtb represents substantial challenges to bacteriologists. Laboratory-associated infections were documented as early as 1960, but a major category of subjects had an unknown origin of transmission. Realizing the seriousness of this finding, in 1969, Arnold Gerhard Wedum [4] introduced bio-safety guidelines and categorized various etiological agents on the basis of bio-hazard (or bio-safety categories 1,2,3, or 4) capability. Mtb was classified as a BSL-3 pathogen, which requires special bio-containment (BSL3) because of its aerosol transmissibility.

Stringent requirements for bio-safety precautions and for the engagement of resources to test large and diverse chemical libraries for bactericidal activity under BSL3 disciplines are major impediments to the discovery of much-needed new mycobactericidal agents. However, alternate options for high-throughput WCS with avirulent mycobacterial surrogates (Msm or *M. bovis* BCG) with MABA or reporter-gene (lux or GFP) strains have been reported with limited success [8–11]. These screens determine the MIC and select only the growth-inhibitory compounds that extrapolate the killing potential of compounds but may or may not kill Mtb at all, unless confirmed to be bactericidal in nature. In fact, none of these screening strategies specifically selects bactericidal compounds. Therefore, the methods used to select bactericidal compounds in the early discovery stages would be of immense value. The true cidality (*vs*. viability) of these compounds can be determined only from cumbersome, agar-based cfu enumeration screens [13].

Here, we report the validation of a high-throughput spot-assay that can directly select bactericidal compounds in BSL3 facilities that comply with all bio-safety guidelines. Although this high-throughput spot-assay does not reduce the incubation time, it can considerably enhance the capacity to screen large compound libraries and select cidal compounds by using quantitative data output. The spot-assay is relatively simpler and works on the principle of viability. Hence, we could develop a robust cidality screen with a panel of RIOE reference drugs. The initial proof-of-concept validation in Msm (a fast-growing saprophyte that does not require BSL3 for handling) enabled multiple studies with more replicates and the rapid incorporation of modifications into the screen. Msm results could be successfully replicated in Mtb, with excellent MBC correlation between the spot-assay and conventional cfu-based readouts. The performance of the spot-assay for Mtb raised no bio-safety concerns.

Furthermore, an excellent correlation in cidality was observed when a set of 250 compounds were tested in Mtb by automated high-throughput spot-assay in parallel with the gold-standard plating method. The assay enormously increased the screening capacity, reduced the Mtb exposure risk, and reduced the additional burden of laborious experiments under BSL3 constraints. A strong positive correlation with excellent QC data, as analyzed by a Manhattan test, further strengthened our confidence in the spot-assay. One of the major problems observed in myco-bacteriology is the translation of MIC to MBC. Even an extensive lead optimization effort may fail to yield MBC-positive compounds. To overcome this challenge, the spot-assay enables the direct selection of bactericidal compounds, which would substantially reduce the attrition of in vivo translation. Our objective was to validate a cidality screen using the spot-assay; hence, we strategically tested a library of 250 ‘known inhibitory compounds’ (single point, at 30μM) on Mtb. Only 1 of 250 compounds lost its inhibition, which might have been caused by solubility issues and was also later reflected in the MBC values. Such minor variations of <10% compounds in early discovery may be attributed to their non-optimized physical properties. Spot-assays could efficiently differentiate static *vs*. cidal compounds in addition to confirm the MIC (Fig. 4a).

A high proportion (189/250 compounds, or 75.6%) of compounds exhibited MBC/cidality because these compounds were selected from the single-point inhibition screen as known inhibitors of Mtb. Of this 75.6% of the MBC-positive group, 91.53% of the compounds overlapped (within a 4-fold variation) in both methods, i.e., the spot and the conventional plating methods. Of the remaining 24.4% (61/250) of the ‘non-MBC category compounds’ from the spot-assay, 90.16% (55/61) of the compounds were consistent across both methods. The spot-assay is simple to perform and lacks any bio-safety concern; hence, spotting an entire dose range also allows for the extensive and comparative grading of bactericidality beyond the 2log_10_ killing. The entire cidality spectrum can be measured by spot-assay. Isoniazid (as 2 QC/plate), which is a selective mycobacterial inhibitor, ruled out any contamination in the entire screening process from the minimal manual interventions involved.

Following the validation of the spot-assay’s ability to handle large compound libraries, we applied a strategy for handling a large number of Mtb strains to perform MBC_90_ studies with a concept similar to that of the MIC_90_. The MBC_90_ studies could be performed comfortably with the advantage that the spot-assay essentially does not require any strain modifications, and it could be performed on any strain/isolate. The spot-assay is convenient, automated and less hazardous under BSL3 constraints, and it does not require any dilutions or plating steps, except for spotting 25μl of culture onto 24-well agar beds. Data enumeration is also possible using closed plates without opening the lids. This setup enabled the comfortable testing of 20 clinical isolates (including drug-resistant isolates) simultaneously in a high-throughput manner. Bio-hazardous aerosol formation (sensitive/MDR/XDR clinical isolates) is reduced substantially, improving individual safety. This report is the first to use the MBC_90_ concept demonstration on highly infectious pathogens such as Mtb.

Furthermore, because the readout for the spot-assay is viability, we could apply it to evaluate Mtb survivors from the cumbersome kinetic screens. The essentiality of drug targets under in-vitro and in-vivo conditions helped to establish the targets’ relevance to the disease. Target modulation using various chemical inhibitors or genetic silencing (antisense RNA, siRNA etc.) has been reported previously by our group [18,16,17]. A good correlation of data from a rifampicin killing kinetic screen in Mtb by the spot-assay *vs*. the conventional cfu assay encouraged us to implement the spot-assay for selecting cidal targets using Mtb gene silencing. The spot-assay could successfully demonstrate the gene-silencing effects and confirm the cidality (*ppk>pyrH*) as well as the static nature of these targets. The resulting target essentiality data are consistent with the published reports [19]. An excellent correlation of survival kinetics trends for spot-assay *vs*. cfu enumeration of 3 genes, namely *ppk*, *pyrH* and *coaD*, corroborated its suitability for measuring the effect of genetic silencing as well. Hence, this validated HTS platform helped in the selection of more vulnerable targets in a simultaneous and reproducible manner. Although this method certainly has advantages for anti-mycobacterial compound screening in BSL-3 facilities, it can, in principle, be applied to any colony-forming organisms (prokaryotes and yeasts). This system has the capacity to test the entire 10c-DR, and a visual (or picture archive) examination of the 24-well MBC plate gives direct information on multiple factors: it simultaneously enables a rapid marking of MBC from a well containing ≥30 colonies, the grading of the equipotent compounds, and a comparison of cidal targets, especially in the kinetic screens. Our studies clearly demonstrate a multipurpose use of the spot-assay in identifying bactericidal compounds and cidal targets in a high-throughput format in BSL3 settings. This assay will have a major impact on the discovery of novel anti-TB therapeutics.

Most importantly, the high-throughput spot-assay is simple, resource saving, and efficient, economical, and ergonomic (EEE) and has the potential to impact the turn-around time (Table 2) for selecting cidal anti-TB compounds/cidal Mtb targets without any bio-safety concerns. The assay will considerably improve the design-make-test-analyze (DMTA) cycle in TB drug discovery, which is a very long process because of the inherent nature of the organism. Moreover, given that many drug discovery programs fail due to a lack of cidal compounds or because of the inability to add cidality following synthesis, the spot-assay may enable the selection of cidal compounds and a faster progression into PK and animal studies.

**Table 2:** A complete automation of spot-assay in the BSL3 facility resulted in increased EEE (Efficiency, Economy and Ergonomics) to reduce the FTEs (resource) and improve the DMTA.

MBC high-throughput method: 24-well format (4 compounds, 10 conc. dose response)						
Method	Time & effort/person	Media supplement	Incubation space	Spread of plates	Throughput/day	Way forward
Conventional agar plate	8 hrs.	10 liters	One incubator	Clutter	**Low** (5 compounds max.)	na
24-well agar plate	**20 min**. (Including labeling, packing etc.)	250–400 ml	Small corner of incubator	Compact	**Very High** (~500 compounds)	Apply to any cfu-based screens

DMTA- Design, Make, Test, Analyze.

## Conclusions

In this manuscript, we provide the first report on the development and validation of a cfu-based high-throughput spot-assay to enumerate pathogens such as *M. tuberculosis* in a BSL3 facility. This high-throughput method is bio-safety-compliant and selects myco-bactericidal compounds in a miniaturized, 24-well format. We answered two critical questions that were instrumental for achieving the desired precision and throughput: **1)** what is the minimum culture volume to spot; and **2)** is there any drug carry-over during this process? The assay was validated with RIOE reference drugs. We were able to select bactericidal compounds in a high-throughput manner from a set of 250 compounds. Furthermore, the application of the spot-assay could determine, for the first time, the MBC_90_ (which is more meaningful than the MIC_90_) on Mtb for sensitive and resistant clinical isolates. The spot-assay described here has the potential to identify not only cidal compounds from large-compound libraries but also cidal targets by AS-RNA gene silencing in kinetic survival screens.

## References

[1] Global Tuberculosis Report (2013) World Health Organization: Geneva p1 Available: http://www.who.int/tb/publications/global_report/gtbr13_main_text.pdf?ua=1.

[2] Wells WF (1934) On airborne infection II: Droplets and droplet nuclei. Am J Hyg 20: 611–618.

[3] Riley RL, Wells WF, Mills CC, Nyka W, Mclean RL (1957) Air Hygiene in tuberculosis: quantitative studies of infectivity and control in a pilot ward. Am Rev Tuberc and Pulm Dis 75: 420–431. 13403171 10.1164/artpd.1957.75.3.420

[4] Wedum AG, Kruse RH, Detrick F, Frederick MD (1969) Assessment of risk of human infection in the microbiological laboratory. Ft. Belvoir Defense Technical Information Center JUL 1969.

[5] Zhang L, Schweizer L (2011) Building the bridge from drug discovery to clinical research using pathway approaches. International Drug Discovery April/May,60–67.

[6] Andries K, Verhasselt P, Guillemont JEG, Gohlmann HWH, Neefs JM, et.al (2005) Diarylquinoline, drug active on the ATP synthase of *Mycobacterium tuberculosis* . Science 307: 223–227. 15591164 10.1126/science.1106753

[7] Beydon MH, Fournier A, Drugeault L, Becquart J (2000) Microbiological high throughput screening: an opportunity for the lead discovery process. J Biomol Screen 5(1):13–22. 10841596 10.1177/108705710000500105

[8] Cooksey RC, Morlock GP, Beggs M, Crawford JT (1995) Bioluminescence method to evaluate antimicrobial agents against *Mycobacterium avium* . Antimicrob Agents Chemother 39: 754–756. 7793886 10.1128/AAC.39.3.754 PMC162618

[9] Collins LA, Torrero MN, Franzblau SG (1998) Green fluorescent protein reporter microplate assay for high-throughput screening of compounds against *Mycobacterium tuberculosis* . Antimicrob Agents and Chemother 42(2): 344–347. 9527783 10.1128/aac.42.2.344 PMC105411

[10] Altaf M, Christopher HM, David SB, O’Toole R (2010) Evaluation of the *Mycobacterium smegmatis* and BCG models for the discovery of *Mycobacterium tuberculosis* inhibitors. Tuberculosis 90: 333–337. 10.1016/j.tube.2010.09.002 20933470

[11] Ruth McNerney Kambashi BS, Kinkese J, Tembwe R, Godfrey FP (2004) Development of a bacteriophage phage replication assay for diagnosis of pulmonary tuberculosis. J Clin Microbiol 42(5): 2115–2120. 10.1128/JCM.42.5.2115-2120 15131178 PMC404598

[12] Primm TP, Franzblau SG (2007) Recent advances in methodologies for the discovery of antimycobacterial drugs. Current Bioactive Compounds 3: 1–7.

[13] Gilchrist JE, Campbell JE, Donnelly CB, Peeler JT, Delaney JM (1973) Spiral plate method for bacterial determination. Applied Microbiology 25(2): 244–252. 4632851 10.1128/am.25.2.244-252.1973 PMC380780

[14] CLSI- Clinical and Laboratory Standards Institute (CLSI), Susceptibility testing of Mycobacteria, Nocardia, and other aerobic actinomycetes 940 West Valley Road, Suite 1400, Wayne, PA 19087 USA. 31339680

[15] Davey H (2011) Life, death and in-between: meanings and methods in Microbiology. Applied and Environmental Microbiology 77(16): 5571–5576. 10.1128/AEM.00744-11 21705550 PMC3165249

[16] Kaur P, Agarwal S, Datta S (2009) Delineating bacteriostatic and bactericidal targets in mycobacteria using IPTG inducible antisense expression. PLOS One 4(6) e5923 10.1371/journal.pone.0005923 19526063 PMC2691988

[17] Jagannathan V, Kaur P, Datta S (2010) Polyphosphate kinase from *M. tuberculosis*: an interconnect between the genetic and biochemical role. PLoS One 5(12): 2143336. 10.1371/journal.pone.0014336 PMC3002279 21179463

[18] Jayaram R, Gaonkar S, Kaur P, Suresh BL, Mahesh BN, et.al (2003) Pharmacokinetic-pharmacodynamic relationships for rifampicin in an aerosol infection model of tuberculosis. Antimicrob Agents and Chemother 47: 2118–2124. 12821456 10.1128/AAC.47.7.2118-2124.2003 PMC161844

[19] Sassetti CM, Boyd DH, Rubin EJ (2003) Genes required for mycobacterial growth defined by high density mutagenesis. Mol Microbiol 48(1): 77–84. 12657046 10.1046/j.1365-2958.2003.03425.x

